# Optimal iron concentrations for growth-associated polyhydroxyalkanoate biosynthesis in the marine photosynthetic purple bacterium *Rhodovulum sulfidophilum* under photoheterotrophic condition

**DOI:** 10.1371/journal.pone.0212654

**Published:** 2019-04-29

**Authors:** Choon Pin Foong, Mieko Higuchi-Takeuchi, Keiji Numata

**Affiliations:** Biomacromolecules Research Team, RIKEN Center for Sustainable Resource Science, Wako, Saitama, Japan; Karl-Franzens-Universitat Graz, AUSTRIA

## Abstract

Polyhydroxyalkanoates (PHAs) are a group of natural biopolyesters that resemble petroleum-derived plastics in terms of physical properties but are less harmful biologically to the environment and humans. Most of the current PHA producers are heterotrophs, which require expensive feeding materials and thus contribute to the high price of PHAs. Marine photosynthetic bacteria are promising alternative microbial cell factories for cost-effective, carbon neutral and sustainable production of PHAs. In this study, *Rhodovulum sulfidophilum*, a marine photosynthetic purple nonsulfur bacterium with a high metabolic versatility, was evaluated for cell growth and PHA production under the influence of various media components found in previous studies. We evaluated iron, using ferric citrate, as another essential factor for cell growth and efficient PHA production and confirmed that PHA production in *R*. *sulfidophilum* was growth-associated under microaerobic and photoheterotrophic conditions. In fact, a subtle amount of iron (1 to 2 μM) was sufficient to promote rapid cell growth and biomass accumulation, as well as a high PHA volumetric productivity during the logarithmic phase. However, an excess amount of iron did not enhance the growth rate or PHA productivity. Thus, we successfully confirmed that an optimum concentration of iron, an essential nutrient, promotes cell growth in *R*. *sulfidophilum* and also enhances PHA utilization.

## Introduction

Polyhydroxyalkanoates (PHAs) are a family of biopolyesters that are produced by a wide variety of microorganisms for the purpose of surviving in unfavorable growth and stress conditions; these molecules act as carbon and energy storage, redox regulators and cryoprotectants [1–3]. PHA is one of the well-known bioplastics (biobased, biodegradable and biocompatible) that has been extensively developed with the aims to overcome problems such as plastic solid wastes, harmful chemical substance leaching and dependence on nonrenewable fossil fuels. These are the main disadvantages of petroleum-derived synthetic plastics [4, 5].

However, the high price of PHAs has made them less competitive compared to conventional synthetic plastics due to the high cost of raw materials used in fermentation and downstream purification steps [6–8]. Strategies are available to reduce the PHA production cost such as utilizing cheap carbon substrates [9–11], better designs of bioreactors and PHA cultivation schemes [12], as well as more efficient PHA recovery methods [13]. Photosynthetic microorganisms could produce their own energy and carbon source by utilizing inexpensive and abundantly available resources such as sunlight and carbon dioxide are potential next-generation microbial cell factories [14–16]. Several studies have reported successful PHA production by photosynthetic bacteria such as cyanobacteria and purple bacteria [17, 18]. Additionally, PHA producers derived from marine bacteria and halophiles could also reduce production costs and lower contamination risk by using seawater as culture media [19–21]. A few research groups have been focusing on marine photosynthetic purple bacteria, which have the additional advantage of the ability to grow and biosynthesize PHA under microaerobic conditions [22, 23]. Ideally, these photosynthetic PHA producers could be the alternative solution for heterotrophic bacteria such as wild-type or engineered strains of *Cupriavidus necator* (*C*. *necator*) H16, *Azohydromonas lata* (formerly known as *Alcaligenes latus*), *Pseudomonas putida* and *Escherichia coli*, which are the main workhorses for large-scale production of PHAs [24–26]. These heterotrophic bacteria lack of photosynthesis ability and thus require expensive carbon supplies to sustain their growth and PHA biosynthesis.

A marine photosynthetic purple nonsulfur bacterium, *Rhodovulum sulfidophilum*, is our target PHA producer, because it has high metabolic versatility [27, 28]. The presence of PHAs in this bacterium was first observed using Sudan Black B staining [27]. It has been previously determined that sodium chloride, vitamins, ammonium chloride, phosphate and carbon sources could affect PHA accumulation and cell growth of *R*. *sulfidophilum* [22, 23]. In this study, we evaluated another nutrient component, iron, which is required in many key biological reactions to support microbial growth and activity [29]. We examined the relationship between cell growth and PHA accumulation in a time-dependent manner by controlling the iron concentrations in a carbon-rich growth medium under microaerobic and photoheterotrophic conditions.

## Results

### Effect of iron on cell growth and PHA accumulation

**Fig 1:**
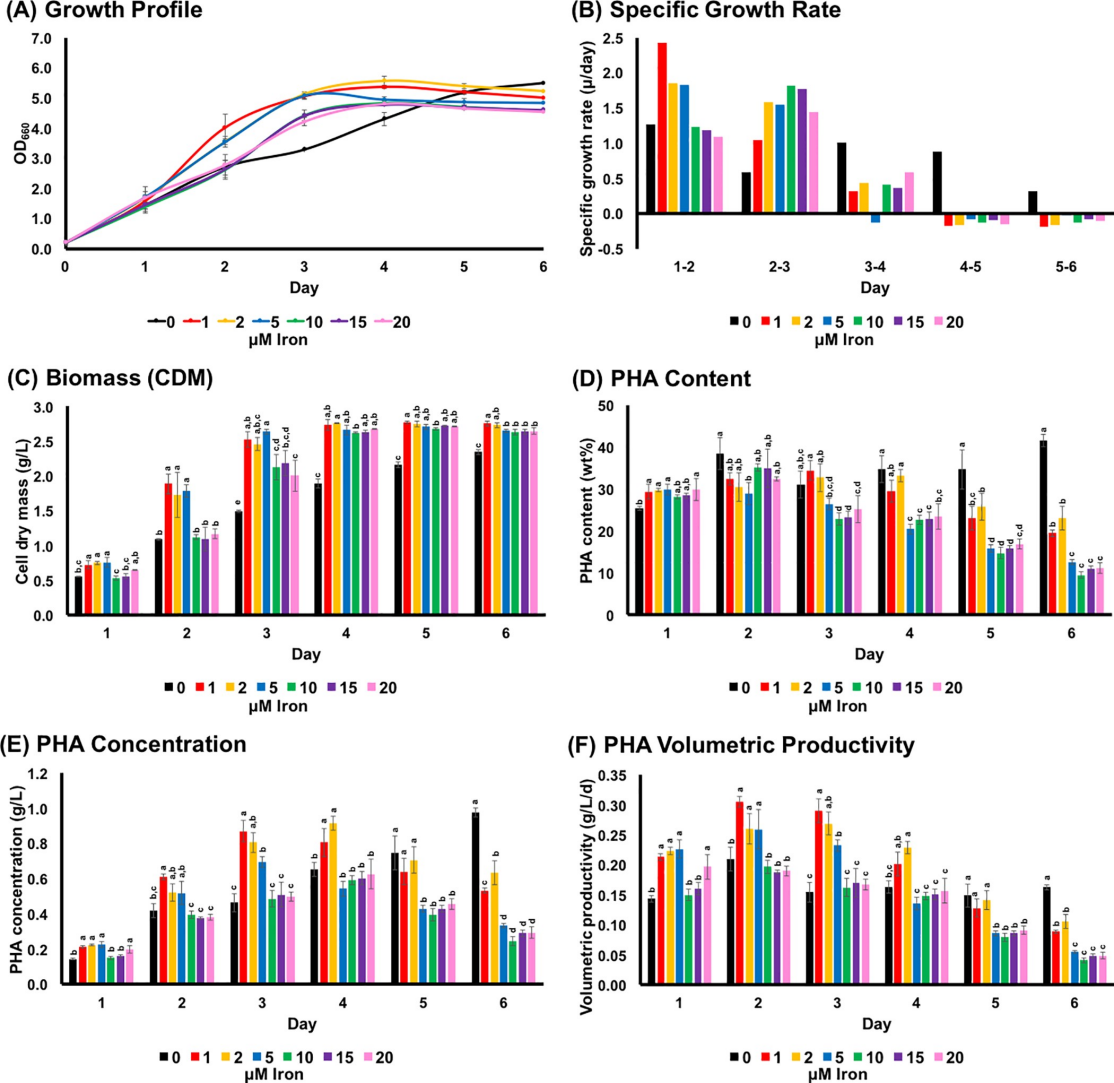
Effect of iron concentration (ferric citrate) on *R*. *sulfidophilum* under microaerobic and photoheterotrophic conditions. (A) cell growth (B) specific growth rate (C) biomass (D) PHA content (E) PHA concentration (F) PHA volumetric productivity. Mean data accompanied by different superscripted letters are significantly different (Tukey’s HSD, p < 0.05).

Addition of 1 to 20 μM of iron in 520-Growth Medium (520-GM) enhanced the growth of *R*. *sulfidophilum* during the first three days of the culturing period compared to the 0 μM iron condition (Fig 1A–1C). In particular, low iron concentrations (1 to 5 μM) had positive effects on cell growth until Day-3, which corresponded to the logarithmic phase. The highest specific growth rate, 2.42 d^-1^ was achieved in the presence of 1 μM iron between Day-1 and Day-2 (Fig 1B). In the presence of iron, the specific growth rates decreased to negative values after Day-4, indicating that the cultures reached stationary phase faster than those in the 0 μM iron condition. An interesting observation was an extended logarithmic phase in the culture with 0 μM iron, even after the six day culture period. The same trend was observed for cell dry mass (CDM) (Fig 1C). CDMs were relatively higher in lower iron concentration conditions until Day-3, while the CDM continued increasing even at Day-6 in cultures not cultivated with iron. The cultures reached maximum CDMs (~2.5 to 2.8 g/L) after entering stationary phase.

It is also worth noting the discrepant results between optical density and CDM (Fig 1A and 1C) for some data points. For instance, there were no significant differences in term of CDM (2.6 to 2.8 g/L) between Day-4 and Day-5 (1 to 20 μM iron), but there were significant differences in OD_660_ (4.6 to 5.6) (S1 Table). Indeed, this was clearly shown in the 0 μM iron culture at Day-6. This discrepancy could be due to the light scattering effect of PHA granules, which decreases the intensity of transmitted light in turbidity measurements [30], as has been shown in *C*. *necator* H16 (wild-type) and its PHB-negative mutant (strain PHB¯4).

On the other hand, the addition of iron compound, namely ferric citrate, did not improve the ability of *R*. *sulfidophilum* to accumulate PHAs in 520-GM (Fig 1D). Indeed, the PHA content gradually decreased in the presence of ≥5 μM iron after Day-2. Furthermore, the PHA content was significantly lower in cultures with ≥5 μM iron after Day-3. The only exception was the culture with 0 μM iron, in which the PHA content gradually increased during the six day culturing period. PHA concentrations were relatively higher in low iron concentration conditions (1 and 2 μM) until Day-4 because of the higher CDMs (Fig 1E). The low PHA contents for cultures with ≥5 μM iron, as shown in Fig 1D, lead to low PHA concentrations after Day-5. Only culture with 0 μM iron showed an increasing trend for PHA concentration because of increases in both CDM and PHA content during the culture period. Regarding PHA volumetric productivity (i.e., PHA concentration per time of culture), *R*. *sulfidophilum* with 1 μM iron achieved the highest productivity of 0.29 g/L/d at Day-2 before the culture entered stationary phase (Fig 1F). The monomer composition of the accumulated PHA for all the experiments in this study was 3-hydrobutyrate (3HB) with negligible or very trace amount (< 1 wt%) of 3-hydroxyvalerate (3HV). Thus, we were unable to detect any change in monomer composition of the PHA.

### Correlation between PHA accumulation and cell growth

**Fig 2:**
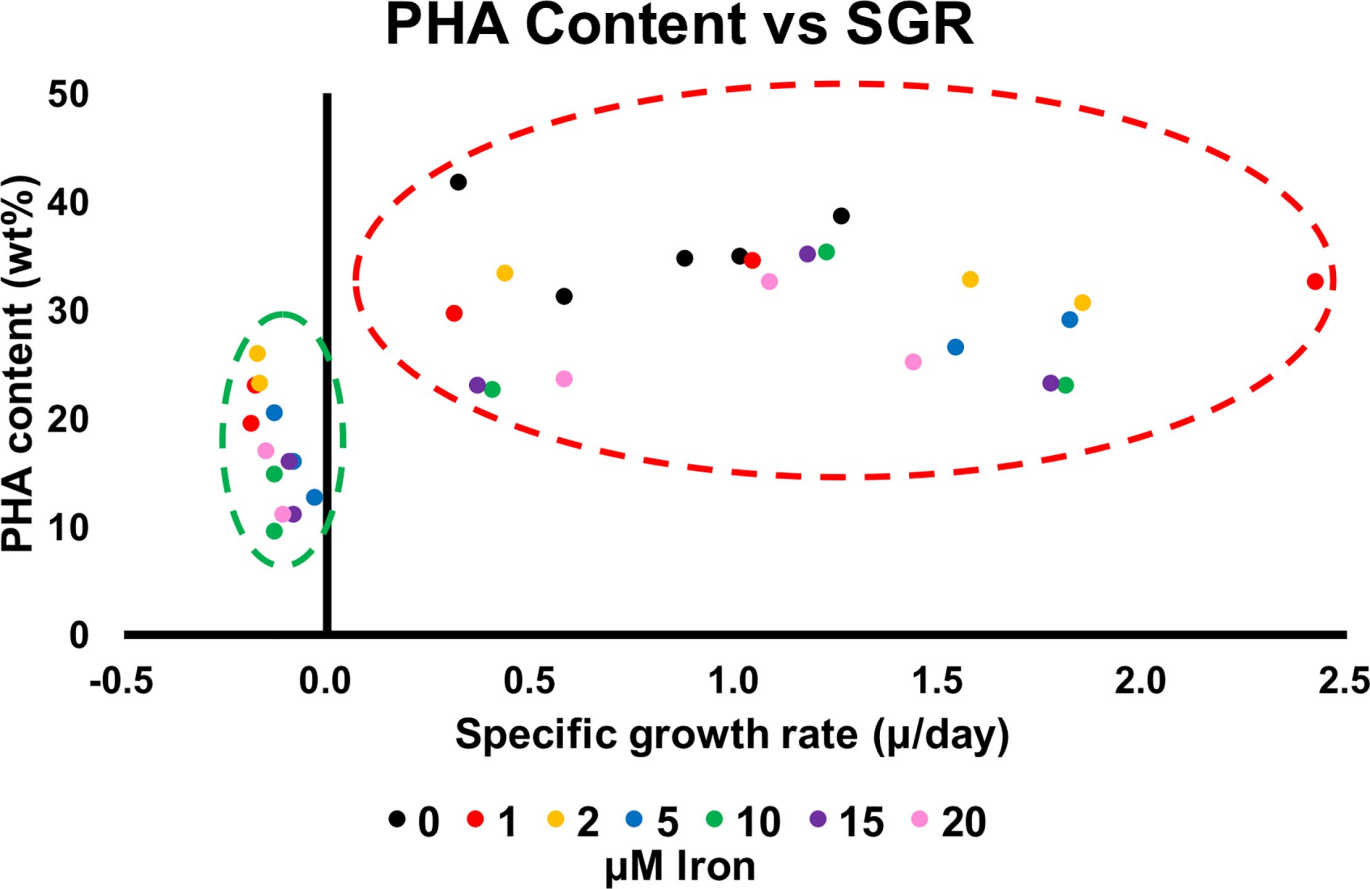
Correlation of PHA content with specific growth rate (SGR). PHA contents were relatively higher during periods of positive SGR (red-dashes circle), while PHA contents were relatively lower during periods of negative SGR (green-dashes circle).

As shown in Fig 1, iron concentrations affected both cell growth and PHA accumulation. However, there was no obvious relationship between iron concentration and the PHA accumulating ability of *R*. *sulfidophilum* in the first two days of culturing period. One of the possible explanations for the trend in PHA accumulation and utilization could be linked to the growth phase of *R*. *sulfidophilum*. PHA contents were high early in the cultivation period and then decreased gradually in the presence of iron (Fig 1D). Culture with 0 μM iron resulted in a continuous increase in CDM and PHA contents (Fig 1C and 1D). These results suggest that PHA was synthesized and accumulated during the logarithmic phase and then was actively utilized by cells during the late logarithmic and stationary phases. We could observe this relationship in the growth profile and in examining the PHA content versus the specific growth rate (Fig 2). PHA contents were relatively higher (>23 wt%) during culture periods with a positive specific growth rate (SGR), but became relatively lower (<23 wt%) during periods with a negative SGR. Indeed, this was clearly shown in the 0 μM iron condition, where the PHA was not utilized by cells during the six day logarithmic phase.

### Two-stages cultivation of *R*. *sulfidophilum*

**Fig 3:**
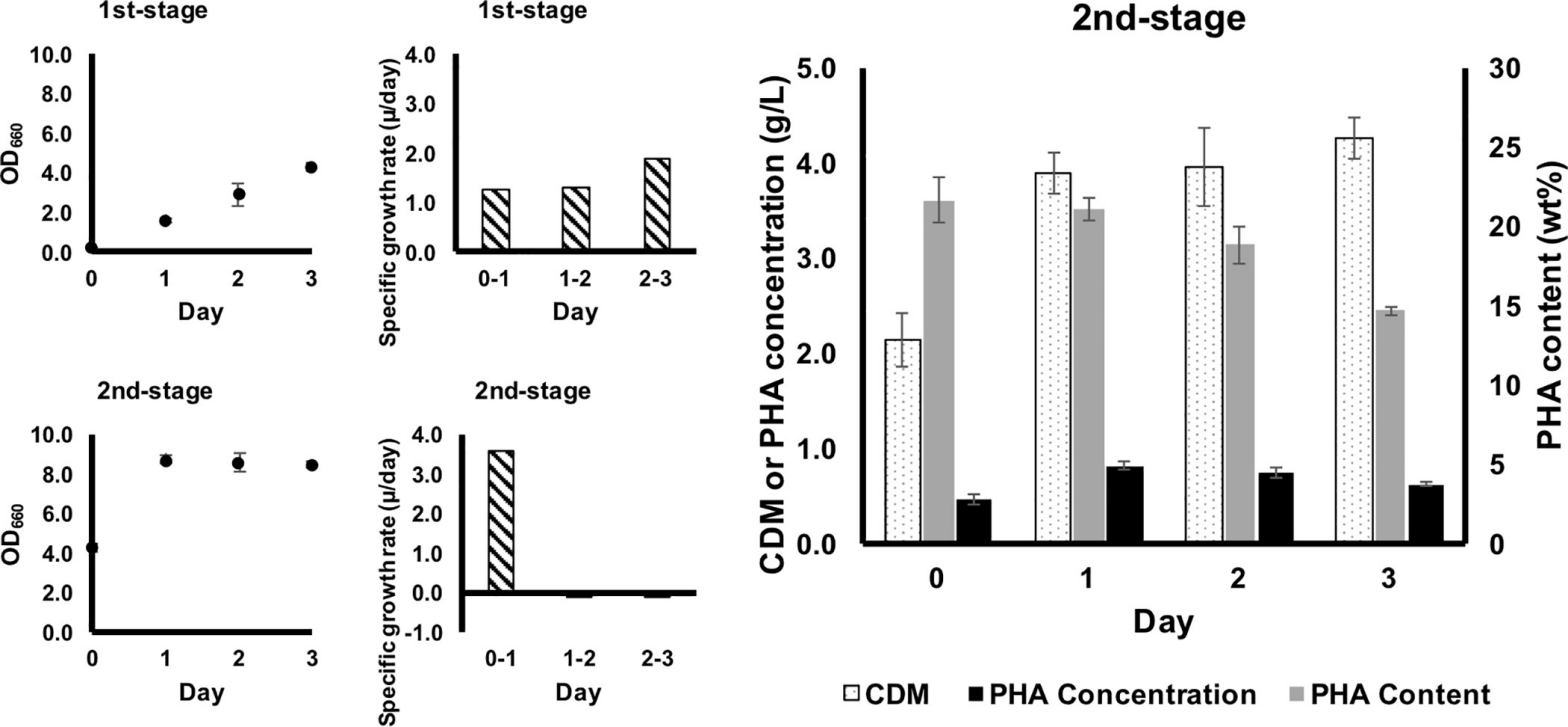
Two-stages cultivation of *R*. *sulfidophilum* under microaerobic and photoheterotrophic conditions. First-stage 520-GM contained 1 μM iron to promote rapid cell growth before reaching stationary growth phase. In second-stage cultivation, cells at Day-3 of first-stage cultivation were harvested by centrifugation and washed once with 2 wt% NaCl solution before added with fresh 520-GM contained 0 μM iron. Biomass (CDM), PHA content and PHA concentration were determined in the second-stage cultivation.

In order to investigate the effect of iron on *R*. *sulfidophilum* during stationary growth phase, two-stages cultivation approach was performed by eliminating iron in the second-stage cultivation (Fig 3). One μM iron was included in the first-stage culture to promote rapid cell growth before the culture reached stationary growth phase at Day-3. The cells were harvested and added with iron-free 520-GM in the second-stage cultivation. Both CDM and OD_660_ increased drastically from 2.14 g/L to 3.89 g/L and from 4.26 to 8.71, respectively, within 24 h cultivation period. However, no changes were observed in PHA content (21 to 22 wt%). Surprisingly, the culture has entered stationary growth phase after 24 h. Both PHA content and PHA concentration were decreased gradually after Day-1 cultivation in the iron-free second-stage culture.

## Discussion

Iron is one of the essential nutrients for major biological reactions and processes such as photosynthesis, nitrogen fixation, respiration, central metabolism and DNA repair [31]. The importance of iron in promoting cell growth of *R*. *sulfidophilum* was clearly shown in this study, where 1 μM iron was found to be optimal to achieve rapid cell growth and cell biomass accumulation in a shorter period of time compared to the 0 μM iron and excess iron conditions (Fig 1A–1C). Notably, the culture in the absence of iron showed the highest amount of PHB per CDM, which is very important parameter affecting cost of downstream processing. On the other hand, high iron concentrations had a negative effect on cell growth in the logarithmic phase. Oversupply of iron could lead to generation of toxic free radicals or reactive oxygen species (ROS) in the cells via the Fenton/Haber-Weiss reaction in the presence of oxygen [32]. This could be the reason for the slower growth and cell biomass accumulation of *R*. *sulfidophilum* in the presence of ≥10 μM iron during the logarithmic phase.

Generally, accumulation of PHAs in most of the PHA-producing bacteria is triggered in conditions of unbalanced nutrients or in the absence of essential nutrients such as nitrogen, sulfate, magnesium, potassium [33], iron [9] [10] [11], phosphorus [34], oxygen [35], but with an excess of a carbon supply. Under these macro- and micro-nutrients deficient conditions, cultures would enter stationary growth phase, where the excessive acetyl-CoA are directed to poly-3-hydroxybutyrate (P3HB) biosynthesis pathway instead of tricarboxylic acid (TCA) cycle [33, 36]. Most of these bacteria synthesize PHA during the late logarithmic or stationary phases and are known as nongrowth-associated PHA producers [37]. However, there are always exceptions where PHA is synthesized during the logarithmic phase in growth-associated PHA producers, such as *Azohydromonas lata* (formerly known as *Alcaligenes latus*) [38]. Previous studies reported that *R*. *sulfidophilum* accumulates PHAs in a growth-associated manner during the logarithmic phase [23], and there is no enhancement of PHA production under nitrogen-limited conditions [22] except for vitamins-free conditions, which contrasts the findings for most of the nongrowth-associated PHA producers [37]. These observations were further supported by the results of this study. Indeed, growth-associated PHA producers are promising candidates for large-scale continuous PHA production (chemostat), which has advantages such as higher productivity, constant product quality and lower production costs [8]. In the continuous PHA production scheme for growth-associated PHA producers, only one chemostat is required instead of two or multiple chemostats in a series, which is necessary in the case of nongrowth-associated PHA production [39]. This is because the media composition for optimum cell growth and PHA accumulation is different among PHA producers.

Significant decreases in PHA content, PHA concentration and PHA volumetric productivity were detected after Day-2 or Day-3 of cultivation in the presence of 1 to 20 μM iron. Additionally, the PHA contents and PHA concentrations were also significantly lower in cultures with ≥5 μM iron after Day-3. These could be explained by the positive correlation between SGR and PHA content (Fig 2), where the accumulated PHA in logarithmic growth phase (positive SGR) was utilized by the culture when the culture entering stationary growth phase (zero or negative SGR). Besides, iron-free condition has demonstrated continuous logarithmic growth and increasing trend of PHA concentration, as well as stable PHA volumetric productivity. Therefore, it may be possible to achieve high cell biomass and also high PHA productivity by conducting two-stages PHA cultivation scheme, where iron was removed in the second-stage culture. As we had expected, high cell biomass could be produced but not in PHA content and PHA concentration (Fig 3). The reason could be because of nutrients depletion within 24 h (in second-stage) due to rapid cell growth and the culture has reached stationary growth phase. Another possibility is hydrogen production. Previous study has shown competition for reduction equivalents during both the PHA and hydrogen production processes and the utilization of PHA as a substrate for hydrogen production in *R*. *sulfidophilum* [40]. Hydrogen evolution is catalyzed by nitrogenase in purple non-sulfur bacteria under nitrogen-deficient condition [41, 42]. Hydrogen was produced in *R*. *sulfidophilum* during the late logarithmic and stationary phases [43]. Further investigations are needed to clarify this competitive relationship for reducing equivalents in *R*. *sulfidophilum*.

In our previous study, we evaluated the expression level of PHA synthase gene (*phaC*) and PHA depolymerase gene (*phaZ*) at the logarithmic growth phase using the cycle threshold values (Ct) of quantitative real-time RT-PCR analysis [22]. The gene expression levels for both *phaC* and *phaZ* were relatively the same at the logarithmic growth phase. This implies that expression level of PHA biosynthesis genes were not altered in the growth stages. However, we did not characterized the detail during the stationary phases. Similar result were shown by a *Pseudomonas putid*a strain, which also demonstrated growth-associated PHA production [44]. PHA operon proteins, including depolymerase, are expressed from the beginning of the growth phase. The *phaZ* gene was expressed throughout the growth cycle. No major changes were observed in transcript levels of *phaC1* and *phaC2*, during the first 24 h of cultivation and this was accompanied by a steady increase in PHA accumulation. At 48 h, correlating with maximum levels of PHA accumulation, a rapid and substantive increase in the transcription of *phaC1* was observed (4.5-fold) and in parallel, increase in *phaZ* transcriptional activity a (6-fold). This was followed by a rapid decrease in the PHA content, and also both *phaC1* and *phaZ* transcript levels.

Chowdhury and coworkers reported that various medium compositions and concentrations could affect PHA accumulation and cell growth in *R*. *sulfidophilum*, such as sodium chloride, vitamins, ammonium chloride, phosphate and carbon sources. Both PHA production and cell growth were stimulated by addition of sodium chloride (3 wt%), ammonium chloride (5 mM) and phosphate [23]. Removal of vitamins also greatly enhanced PHA accumulation but had no obvious effect on cell growth. In this study, we successfully confirmed that iron is another essential nutrient that promotes cell growth in *R*. *sulfidophilum* and also enhances PHA utilization during the stationary phase.

Optimum iron concentrations have been shown to be important to enhance PHA production. In Thakor et al (2003) [45], 40 mg/L ferric chloride was found to be optimum for PHB accumulation in *Comamonas testosterone*. Iron concentrations also affected PHA production in mixed culture systems [46, 47]. Thus, an optimum concentration of iron needs to be determined in order to achieve the highest PHA productivity. In summary, we conclude that 1 to 2 μM of iron in 520-GM was able to achieve the highest PHA volumetric productivity of 0.26 to 0.29±0.02 g/L/d at Day-2 and Day-3 of cultivation (Fig 1F). Additionally, when we compared PHA yield (g of PHA per g of utilized carbon substrate) with previous report 0.05 g/g (carbon source: sodium acetate; CDM: 0.42 g/L; PHA content: 56.1 wt%) [23], our 520-GM supplemented with 1 μM iron could generate a much higher PHA yield 0.10 g/g (carbon source: sodium malate and sodium pyruvate; CDM: 1.89 g/L; PHA content: 32.4 wt%).

We estimated costs of the media in *C*. *necator* and *R*. *sulfidophilum* (S2 Table). In case of *C*. *necator*, seed culture is nutrient-rich (NR) medium which consists of meat extract, polypeptone, and yeast extract. While PHA-producing medium is nitrogen-limited minimal medium which consists of KH_2_PO_4_, Na_2_HPO_4_, MgSO_4_, NH_4_Cl and trace element solution, carbon source can be either fructose or plant oils. Seed culture and PHA producing medium is 520-GM in *R*. *sulfidophilum*. At the current stage of research progress, *R*. *sulfidophilum* may be not a cost-effective PHA producer compared to heterotroph PHA producers. However, we can further reduce the PHA production cost in *R*. *sulfidophilum* by modifying the 520-GM, for example, vitamin can be removed from the 520-GM, as reported by Chowdhury et al 1996, vitamin-free condition could enhance PHA accumulation [23]. Besides, the carbon sources can be replaced by sodium acetate (cheaper than sodium malate or sodium pyruvate) which is also the best carbon substrate for both cell growth and PHA accumulation [23].

In addition, we have tried to cultivate *R*. *sulfidophilum* under fully photoautotrophic condition with different iron concentrations. As shown in S2 Fig, *R*. *sulfidophilum* did not grow well under fully photoautotrophic condition (520-GM without any carbon sources but supplemented with 1.0 g/L sodium bicarbonate). OD_660_ values were below 0.1 from Day-1 to Day-6. The growth of *R*. *sulfidophilum* under fully heterotrophic condition (without light source) in 520-GM with 1 μM iron, we could obtain about 0.5 g/L of CDM and 5 wt% of PHA, which is not suitable condition for cell growth and PHA accumulation. We previously examined growth of *R*. *sulfidophilum* using artificial seawater under photoheterotrophic condition [22]. PHA accumulation could be induced by adding sodium acetate into artificial seawater medium. If we can use natural seawater instead of freshwater in medium preparation, this would further reduce the PHA production cost.

## Materials and methods

### Media compositions and culture conditions

The marine phototrophic purple nonsulfur bacterium *Rhodovulum sulfidophilum* [27, 48] DSM1374/ATCC35886/W4 was obtained from the American Type Culture Collection (ATCC) biological resource center (BRC). For general cultivation purpose, *R*. *sulfidophilum* was maintained on marine agar or marine broth (BD Difco, New Jersey, USA) under microaerobic condition at 30°C with continuous far-red LED light (730 nm, 20 to 30 Wm^-2^). A different rich medium, 520-Growth Medium (520-GM), was used for PHA accumulation purpose. 520-GM is recommended as growth medium for *R*. *sulfidophilum* by Japan Collection of Microorganisms (https://www.jcm.riken.jp/cgi-bin/jcm/jcm_grmd?GRMD=520). The 520-GM contained the following components per liter: 0.5 g KH_2_PO_4_, 0.25 g CaCl_2_⋅2H_2_O, 3.0 g MgSO_4_⋅7H_2_O, 0.68 g NH_4_Cl, 20 g NaCl, 3.0 g sodium L-malate, 3.0 g sodium pyruvate, 0.4 g yeast extract, 2 mg vitamin B12 and micronutrients including 70 μg ZnCl_2_, 100 μg MnCl_2_⋅4H_2_O, 60 μg H_3_BO_3_, 200 μg CoCl_2_⋅6H_2_O, 20 μg CuCl_2_⋅2H_2_O, 20 μg NiCl_2_⋅6H_2_O and 40 μg Na_2_MoO_4_⋅2H_2_O. The pH of the medium was adjusted to 7.0 before autoclave sterilization.

Different concentrations of ferric citrate, ranging from 0 to 5.0 mg/L (1 to 20 μM of iron) were added into 520-GM to evaluate the effects on cell growth and PHA accumulation. A one-stage cultivation strategy was employed for PHA production, where the *R*. *sulfidophilum* was first precultured in 520-GM until the OD_660_ value reached approximately 2.0 (logarithmic phase) before being transferred to 520-GM with a final OD_660_ value of 0.2 after dilution. A total of 15 mL 520-GM in a 15 mL conical centrifuge tube was used, and the cultures were incubated for six days under microaerobic conditions at 30°C with continuous far-red LED light (730 nm, 20 to 30 Wm^-2^). Cells were harvested by centrifugation at 9,000 × *g* for 10 min, washed with distilled water once, kept at –80°C overnight and then freeze-dried for 24 h.

### Growth profile and cell biomass analyses

The growth profile of *R*. *sulfidophilum* was measured using a UV/Vis-spectrophotometer at an absorbance of 660 nm with a 24-h interval. Specific growth rates (μ/day) were calculated based on the slope of the growth curve [49]. Total cell biomass (g/L) was determined based on cell dry mass of the samples.

### PHA quantification

The PHA content characterization method was modified slightly from a previous study [50]. Approximately 2 mg of lyophilized cells were subjected to methanolysis [1 mL chloroform and 1 mL methanolysis solution (methanol:sulfuric acid, 85:15 v/v)] at 100°C for 140 min. After cooling to room temperature, 1 mL of phosphate buffer (pH 8) was added to the reaction mixture, which was then vortex-mixed and neutralized with 0.5 N NaOH. The lower chloroform layer was transferred to a glass tube containing sodium sulfate anhydrous to remove water content. The PHA content and composition were determined using gas chromatography-mass spectrometry (GC-MS) machine (GCMS-QP2010 Ultra, Shimadzu, Kyoto, Japan) equipped with a 30 m × 0.25 mm DB-1 capillary gas chromatography column (Agilent Technologies, CA, USA). For analysis, a 1 μL volume of sample was injected with helium as a carrier gas (3.30 mL min^-1^). The following temperature program was used to separate ethyl esters: 45°C for 1 min, temperature ramp of 7°C per min to 117°C. The interface and ion source temperatures were 250°C and 230°C, respectively. PHB quantification calibration curve is attached in S1 Fig.

### Statistical analysis

The Statistical Package for the Social Sciences (SPSS) software version 22 (IBM Corp. Released 22.0.0.0, New York, USA) was used for all analyses. Statistically significant differences between groups were determined by one-way analysis of variance (ANOVA) assessments with Tukey post hoc tests, where a p-value <0.05 indicated a significant difference.

## Supporting information

S1 Fig PHB quantification calibration curve. (TIFF)

S2 Fig Time-course of growth under photoautotrophic conditions. Seed culture is normal 520-GM with carbon sources. After the culture has reached OD_660_ ~2.0, it was transferred into 520-GM without any carbon sources but supplemented with 1 g/L NaHCO_3_ for photoautotrophic growth condition.
(TIF)

S1 Table OD660 values or growth of *R. sulfidophilum* in different iron concentrations. Mean data accompanied by different superscripted letters are significantly different on the same day (Tukey’s HSD, p < 0.05).
(PPTX)

S2 Table Most costly components in the media for *C. necator* and *R. sulfidophilum*. (PPTX)
