# Supplementary figures and images for: Optimal iron concentrations for growth-associated polyhydroxyalkanoate biosynthesis in the marine photosynthetic purple bacterium Rhodovulum sulfidophilum under photoheterotrophic condition
Source: PLoS One. 2019 Apr 29;14(4):e0212654. doi: 10.1371/journal.pone.0212654 (PMC6488045; doi:10.1371/journal.pone.0212654)

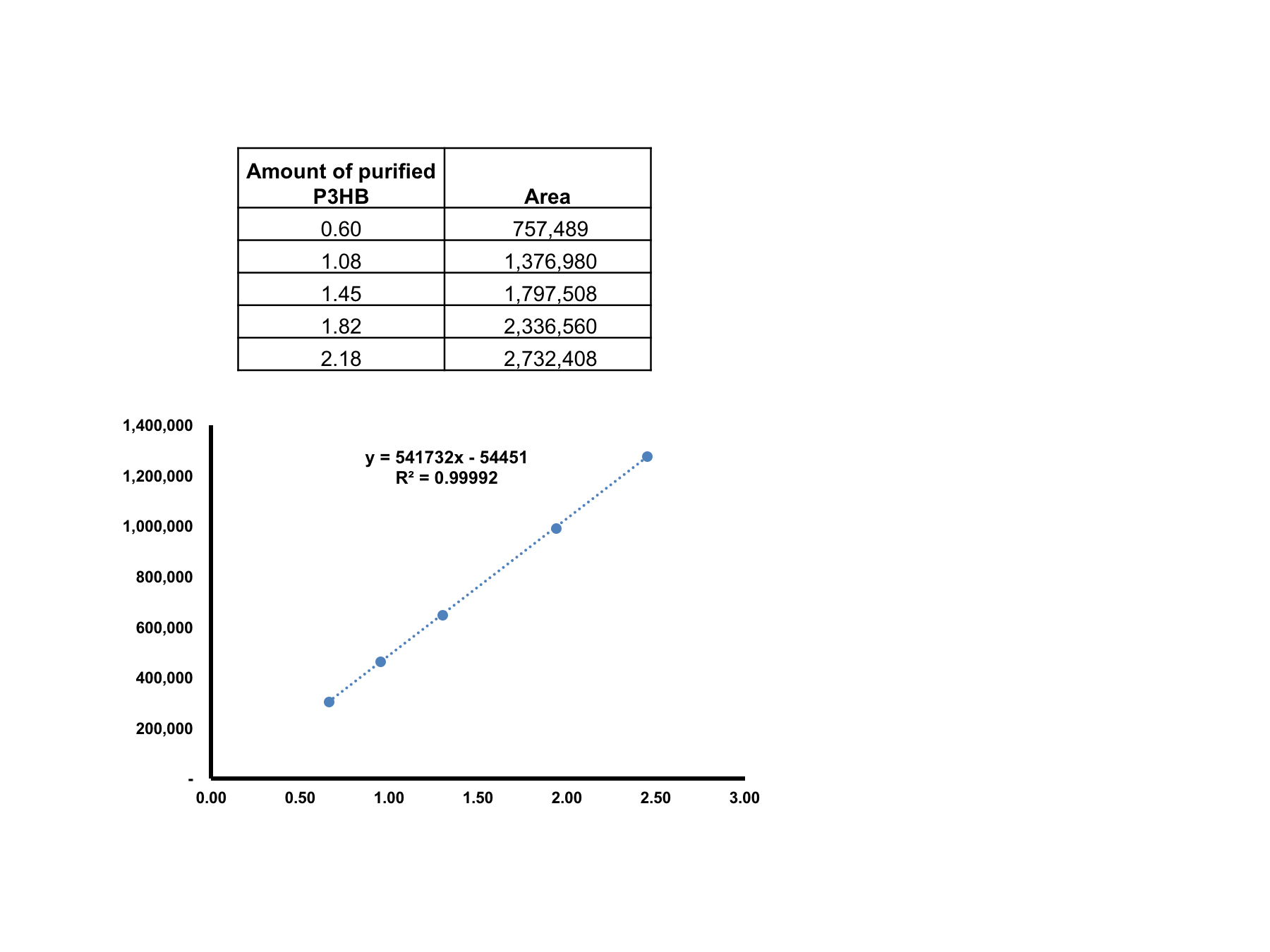

Supplement: S1 Fig — (TIFF) [file pone.0212654.s001.tiff]

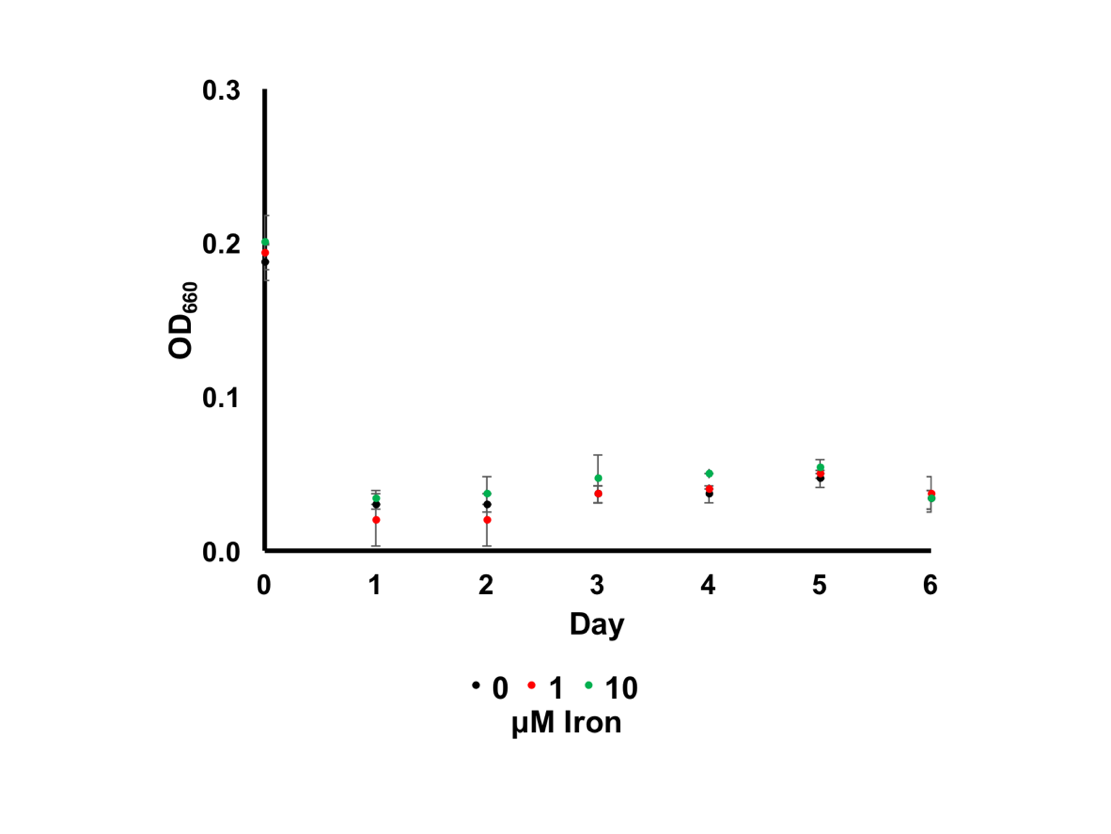

Supplement: S2 Fig — Seed culture is normal 520-GM with carbon sources. After the culture has reached OD660 ~2.0, it was transferred into 520-GM without any carbon sources but supplemented with 1 g/L NaHCO3 for photoautotrophic growth condition. (TIF) [file pone.0212654.s002.tif]
