# Supplementary material for: Optimal iron concentrations for growth-associated polyhydroxyalkanoate biosynthesis in the marine photosynthetic purple bacterium Rhodovulum sulfidophilum under photoheterotrophic condition
Source: PLoS One. 2019 Apr 29;14(4):e0212654. doi: 10.1371/journal.pone.0212654 (PMC6488045; doi:10.1371/journal.pone.0212654)
Supplement: S1 Table — Mean data accompanied by different superscripted letters are significantly different on the same day (Tukey’s HSD, p < 0.05). (PPTX) [file pone.0212654.s003.pptx]

## Slide 1
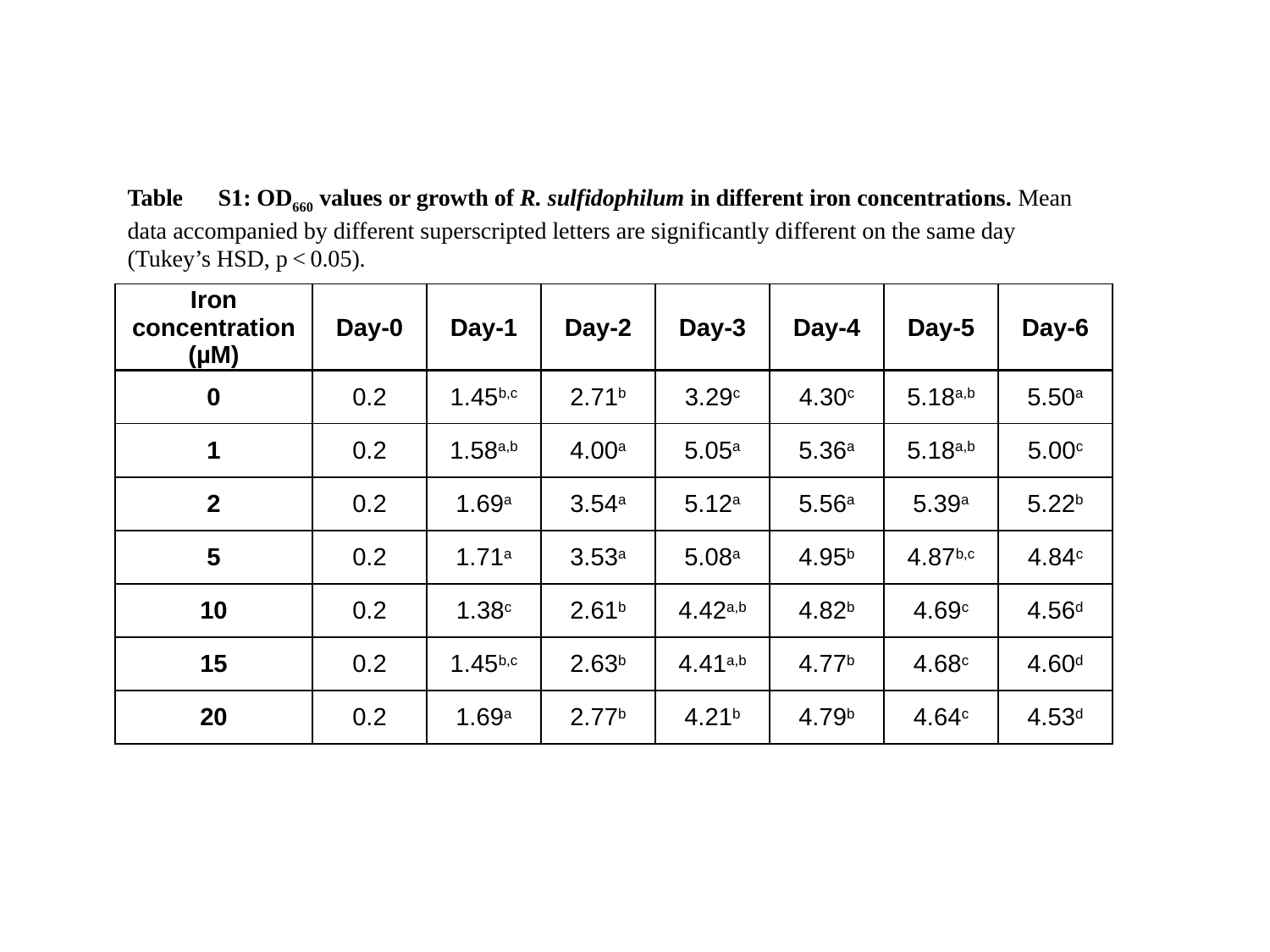

Table　S1: OD660 values or growth of R. sulfidophilum in different iron concentrations. Mean data accompanied by different superscripted letters are significantly different on the same day (Tukey’s HSD, p < 0.05).
| Iron concentration (µM) | Day-0 | Day-1 | Day-2 | Day-3 | Day-4 | Day-5 | Day-6 |
| --- | --- | --- | --- | --- | --- | --- | --- |
| 0 | 0.2 | 1.45b,c | 2.71b | 3.29c | 4.30c | 5.18a,b | 5.50a |
| 1 | 0.2 | 1.58a,b | 4.00a | 5.05a | 5.36a | 5.18a,b | 5.00c |
| 2 | 0.2 | 1.69a | 3.54a | 5.12a | 5.56a | 5.39a | 5.22b |
| 5 | 0.2 | 1.71a | 3.53a | 5.08a | 4.95b | 4.87b,c | 4.84c |
| 10 | 0.2 | 1.38c | 2.61b | 4.42a,b | 4.82b | 4.69c | 4.56d |
| 15 | 0.2 | 1.45b,c | 2.63b | 4.41a,b | 4.77b | 4.68c | 4.60d |
| 20 | 0.2 | 1.69a | 2.77b | 4.21b | 4.79b | 4.64c | 4.53d |
