# Supplementary material for: Optimal iron concentrations for growth-associated polyhydroxyalkanoate biosynthesis in the marine photosynthetic purple bacterium Rhodovulum sulfidophilum under photoheterotrophic condition
Source: PLoS One. 2019 Apr 29;14(4):e0212654. doi: 10.1371/journal.pone.0212654 (PMC6488045; doi:10.1371/journal.pone.0212654)
Supplement: S2 Table — (PPTX) [file pone.0212654.s004.pptx]

## Slide 1
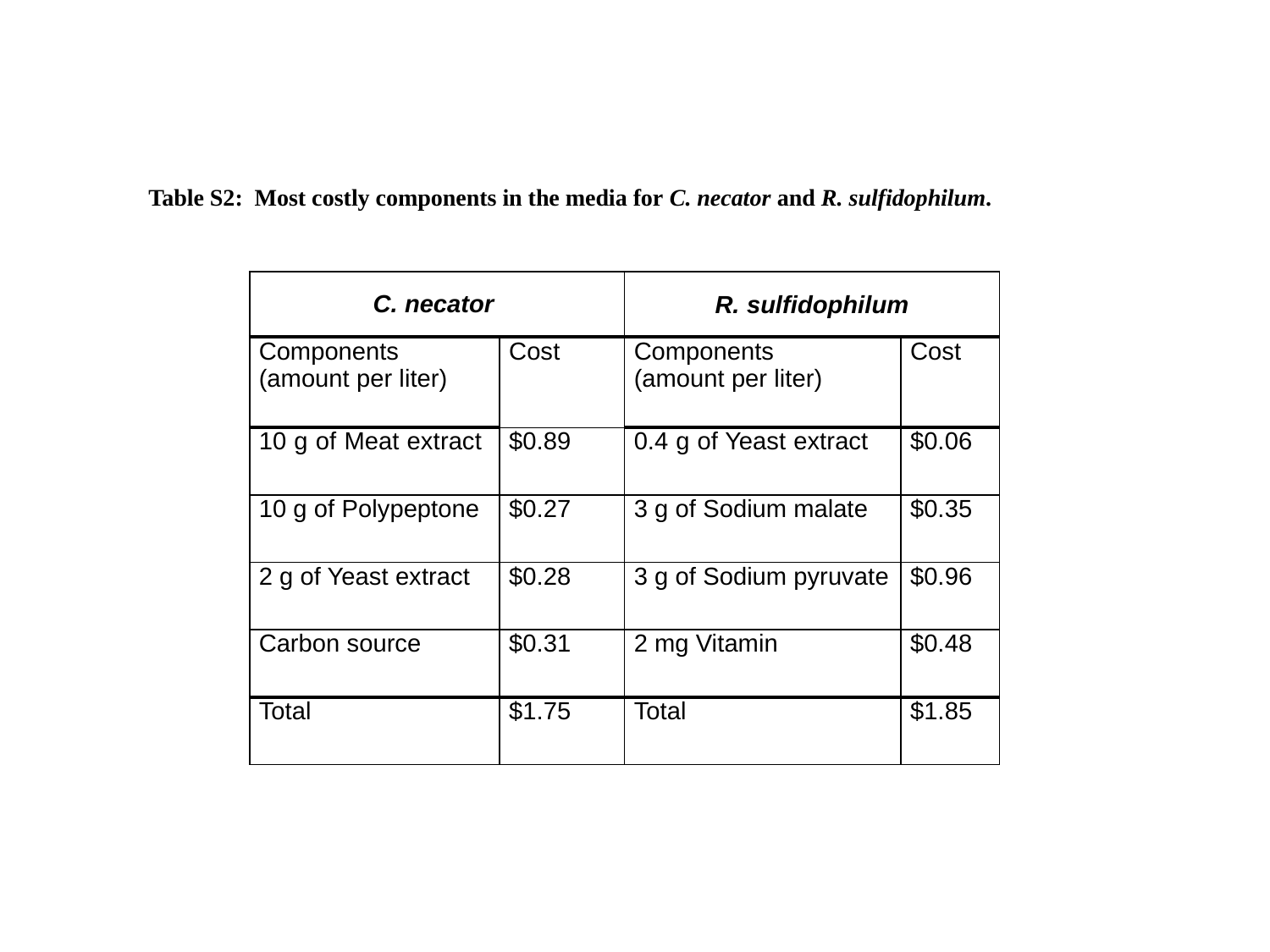

Table S2: Most costly components in the media for C. necator and R. sulfidophilum.
| C. necator | | R. sulfidophilum | |
| --- | --- | --- | --- |
| Components (amount per liter) | Cost | Components (amount per liter) | Cost |
| 10 g of Meat extract | $0.89 | 0.4 g of Yeast extract | $0.06 |
| 10 g of Polypeptone | $0.27 | 3 g of Sodium malate | $0.35 |
| 2 g of Yeast extract | $0.28 | 3 g of Sodium pyruvate | $0.96 |
| Carbon source | $0.31 | 2 mg Vitamin | $0.48 |
| Total | $1.75 | Total | $1.85 |
